# Inter-Population Genetic Diversity and Clustering of *Merozoite Surface Protein-1 (pkmsp-1)* of *Plasmodium knowlesi* Isolates from Malaysia and Thailand

**DOI:** 10.3390/tropicalmed8050285

**Published:** 2023-05-20

**Authors:** Naqib Rafieqin Noordin, Yee Ling Lau, Fei Wen Cheong, Mun Yik Fong

**Affiliations:** Department of Parasitology, Faculty of Medicine, Universiti Malaya, Kuala Lumpur 50603, Malaysia; naqibrafieqin.nr@gmail.com (N.R.N.);

**Keywords:** *Plasmodium knowlesi*, merozoite surface protein-1, genetic diversity, clustering, block IV

## Abstract

The genetic diversity of *pkmsp-1* of Malaysian *Plasmodium knowlesi* isolates was studied recently. However, the study only included three relatively older strains from Peninsular Malaysia and focused mainly on the conserved blocks of this gene. In this study, the full-length *pkmsp-1* sequence of recent *P. knowlesi* isolates from Peninsular Malaysia was characterized, along with Malaysian Borneo and Thailand *pkmsp-1* sequences that were retrieved from GenBank. Genomic DNA of *P. knowlesi* was extracted from human blood specimens and the *pkmsp-1* gene was PCR-amplified, cloned, and sequenced. The sequences were analysed for genetic diversity, departure from neutrality, and geographical clustering. The *pkmsp-1* gene was found to be under purifying/negative selection and grouped into three clusters via a neighbour-joining tree and neighbour net inferences. Of the four polymorphic blocks in *pkmsp-1*, block IV, was most polymorphic, with the highest insertion–deletion (indel) sites. Two allelic families were identified in block IV, thereby highlighting the importance of this block as a promising genotyping marker for the multiplicity of infection study of *P. knowlesi* malaria. A single locus marker may provide an alternate, simpler method to type *P. knowlesi* in a population.

## 1. Introduction

Malaria remains a major public health challenge. In 2021, 247 million people were afflicted by this disease globally, with more than 600,000 deaths reported. Since 2018, Malaysia has had no reports of indigenous malaria cases caused by human malaria parasite species. However, zoonotic malaria caused by *Plasmodium knowlesi* has become the main cause of malaria cases in Malaysia, with a notable increase in the number of cases from 1600 to over 4000 between 2016 and 2018. In 2021, 13 deaths were reported due to this zoonotic infection [1], with 409 cases in Peninsular Malaysia and 3166 cases in Malaysian Borneo (Ministry of Health Malaysia, unpublished data). The situation is similar to other countries in Southeast Asia (SEA), as an upsurge of *P. knowlesi* cases in humans was also reported in neighbouring countries such as Brunei [2], Thailand [3], and Indonesia [4]. No later than 2018, Laos also documented their first human knowlesi case [5], making all countries in SEA endemic to this zoonotic infection, except for Timor-Leste [6]. This quotidian apicomplexan parasite undergoes the shortest asexual life cycle of 24 h in comparison to other human *Plasmodium* species [7], which results in the parasitaemia level increasing rapidly upon infection. High parasitaemia in patients with knowlesi malaria has been correlated with the development of debilitating symptoms and severe cases with potentially fatal outcomes. The fatality rate is similar to malignant malaria caused by *P. falciparum*, at approximately 6% to 9% [8]. Hyperparasitaemia, jaundice, and severe acute kidney injuries are among the clinical presentations and known predictors of severe human knowlesi infection [9,10]. Recently, a histological examination also demonstrated the ring stage of *P. knowlesi* on renal biopsies obtained from patients with acute kidney injury [11]. The microvascular injury may suggest the vascular involvement of the parasite.

A population-based case-controlled study in Sabah, Malaysia previously showed that males were 2.71 times more prone to acquiring *P. knowlesi* malaria compared to females. Other risk factors include staying overnight in the forest, not using mosquito repellent, and a history of malaria infection [12]. Besides that, *P. knowlesi* transmission is driven by natural and human-induced environmental variations, as seen in Sabah, where the shrinking primary forest coverage has been linked to an increase in infections [13]. The shift of monkey population into human settlements and remaining forest patches when habitats are deforested increases the chance of close contact between humans and the monkey hosts. Besides having them as pets, it has also been shown that monkeys present in areas where human activities occur can pose a risk to humans [12]. Asymptomatic infection poses another challenge to eradicating *P. knowlesi*, as it can infect mosquitoes that sustain the transmission [14]. In Malaysia, several studies have reported asymptomatic and/or submicroscopic knowlesi malaria cases [9,15,16], thus raising concerns, as humans can now act as infectious reservoirs and are unaware of harbouring the parasite. Therefore, there is an urgent need to reinforce monitoring and control programs, in addition to conducting active research on developing novel vaccine candidates to mitigate *P. knowlesi* infection.

The merozoite surface protein-1 (MSP-1), one of the most abundant surface proteins of *Plasmodium* spp., is involved in the invasion and survival of the parasite in host erythrocytes. A successful invasion into the host’s erythrocytes is governed by five crucial steps: complex recognition, initial attachment, re-orientation of merozoite’s apical end, formation of irreversible tight junction, and invagination of merozoite into the host’s erythrocyte [17]. MSP-1 mediates the initial attachment of merozoite onto the host’s erythrocyte along with other protein families [18]. In *P. falciparum*, MSP-1 is synthesized upon schizogony and closely linked with the merozoite surface protein-7 (MSP-7) [19], which will form a complex that acts as a ligand and binds to the membrane of the erythrocyte, which is then anchored by a glycosylphosphatidylinositol (GPI). Merozoite surface protein-6 (MSP-6) is also a unit of the MSP complex during the initial attachment [20]. MSP-1 is 185–225 kDa in size, and undergoes dual-endoproteolytic processes during the invasion to produce four protein subunits: 83-, 30-, 38-, and C-terminal 42-kDa. A second process then cleaves the 42-kDa into 33- and 19-kDa subunits [21]. These processes occur not only in the four human *Plasmodium* species, but also in *P. knowlesi* [22]. The 33-kDa subunit detaches and sheds along with the soluble subunits of the first proteolytic processing, while the 19-kDa is invaginated into the host’s erythrocytes [21,23]. Several studies revealed that specific antibodies raised against the C-terminal 42-kDa (MSP-1_42_) inhibited parasite invasion and/or interrupted parasite growth [24,25], thereby demonstrating the importance of this subunit. Furthermore, purifying/negative selection was observed recently within the MSP-1_42_ of *P. knowlesi* isolates from Malaysia [26,27] and Thailand [27], thus suggesting the functional constraint of this subunit.

Polymorphic blocks within the *msp-1* and *msp-2* have been used as markers for genetic diversity and multiplicity of infection (MOI) studies in *Plasmodium falciparum* and *Plasmodium vivax* populations [28,29,30,31,32,33,34], but there have been no reports for *P. knowlesi.* The structure of the *pkmsp-1* gene was reported previously for Thailand *P. knowlesi* isolates [35], thus revealing a mosaic pattern with five conserved blocks (I, III, V, VII, and IX), which are intercalated by four polymorphic blocks (II, IV, VI, and VIII). A recent study investigated the genetic diversity of *pkmsp-1* from Malaysian isolates [27] but focused mainly on the conserved blocks. Furthermore, the study only used three relatively older Peninsular Malaysia strains: H, MR4, and Pk1A. In this study, the full-length *pkmsp-1* of recent *P. knowlesi* isolates from Peninsular Malaysia was analysed, along with *pkmsp-1* sequences from Thailand and Malaysian Borneo that were retrieved from GenBank.

## 2. Materials and Methods

### 2.1. Ethical Clearance

The study received ethical clearance from the Medical Research Subcommittee of the Malaysian Ministry of Health (NMRR-15-67223975) for the use of blood specimens obtained from hospitals and district health offices in Peninsular Malaysia.

### 2.2. Plasmodium Species Confirmation

Twenty previously confirmed *P. knowlesi* human blood specimens by microscopy were re-confirmed via nested PCR targeting the *18S* rRNA gene as described previously [36,37]. The specimens were also screened for the presence of the four human *Plasmodium* species, and none of the specimens had mixed infection. The details of the specimens are listed in Supplementary File (Table S1).

### 2.3. DNA Extraction

*Plasmodium* genomic DNA was extracted from 100 µL of human knowlesi malaria patient blood using Qiagen DNeasy Blood and Tissue Kits (Qiagen, Hilden, Germany) according to the manufacturer’s protocol and eluted with 50 µL of AE buffer in the kit for a total of 2 eluates. The eluates were pooled, and the DNA was stored at −20 °C until further use.

### 2.4. Amplification of pkmsp-1 Gene

The *pkmsp-1* gene was amplified using gene-specific primer pairs PkMSP1-F: 5′ CGTTGGCCACTTTTAAG 3′ and PkMSP1-R: 5′ AATGTGCAGCCAAAGCC 3′ with the following composition: 4.0 µL DNA template, 1X GoTaq^®^ Long PCR Master Mix (Promega, Madison, WI, USA), and 0.4 µM of each primer in 25 µL final volume reaction. The cycling conditions were set at 95 °C for 3 min, then 35 cycles at 94 °C for 30 s, 58 °C for 30 s, and 68 °C for 7 min, followed by a final extension at 72 °C for 10 min. Amplicons were electrophoresed on a 0.7% agarose gel pre-stained with SYBR^®^ Safe DNA gel stain (Invitrogen, Eugene, OR, USA) at 90 V for 40 min and visualised using Gel Doc XR+ System (BioRad, Hercules, CA, USA).

### 2.5. Cloning and Sequencing of pkmsp-1

The amplicons were purified using the QIAquick PCR purification kit (Qiagen, Hilden, Germany) per manufacturer’s protocol, and the purified amplicons were ligated into pGEM-T^®^ TA cloning vector (Promega, WI, USA). The ligation products were then transformed into One Shot™ *Escherichia coli* TOP10F’ competent cells (Invitrogen, Eugene, OR, USA). Colony PCR was then conducted against the transformants using the universal M13 forward (40 mer) and M13 reverse (48 mer) after a 16-h incubation at 37 °C. Plasmids containing the *pkmsp-1* insert were harvested from positive recombinant clones using the QIAprep Spin Miniprep kit (Qiagen, Hilden, Germany) and sent to a commercial laboratory (First BASE Laboratories Sdn. Bhd., Seri Kembangan, Malaysia) for nucleotide sequencing. Four primer pairs (Table 1) were used to sequence the *pkmsp-1* insert via Sanger dideoxy sequencing method.

### 2.6. Genetic Diversity and Natural Selection Analysis

All raw sequences were trimmed to remove pGEM-T^®^ TA vector sequences using BioEdit ver 7.2. The *pkmsp-1* sequences (n = 20) (GenBank accession numbers: ON926538-ON926551 and ON926557-ON926562) were then aligned with the Malaysian Borneo sequences (n = 20) (GenBank accession numbers: ERR274221, ERR274222, ERR274224, ERR274225, ERR366425, ERR985377-ERR985381, ERR985387-ERR985391, ERR2762859, ERR2762860, ERR2762864, ERR2762867, and ERR2762887), Thailand sequences (n = 23) (Genbank accession numbers: JF837339-JF837353 and JX046791-JX046798), *P. knowlesi* strain H (Genbank accession number: PKNH_0728900) and strain MR4-H (Genbank accession number: SAMN04009580) using the ClustalW program in BioEdit ver 7.2. A total of 65 *pkmsp-1* sequences were analysed in this study. Block-wise analyses on the single nucleotide polymorphisms (SNPs), insertion-deletion (indels), number of haplotypes (H), nucleotide diversity (π), and haplotype diversity (Hd) were performed using DnaSP ver 6.12. Departure from neutrality was investigated using Tajima’s D, Fu and Li’s D*, and Fu and Li’s F* tests. Tajima’s D is expected to be 0 when neutral. Both the sliding window plot and the natural selection analyses were conducted using DnaSP ver 6.12. The rate of synonymous (dS) and nonsynonymous (dN) substitutions was determined and evaluated for natural selection using MEGA X. A negative value (dN − dS < 0) signifies purifying/negative selection, while a positive value (dN − dS > 0) signifies diversifying/positive selection [38].

### 2.7. Phylogenetic Inference

The deduced pkmsp-1 amino acid sequences were used to construct a neighbour-joining tree using the Nei–Gojobori (Jukes–Cantor correction) method with 1000 bootstraps in MEGA X. The *P. falciparum* MSP-1 (GenBank accession number: XP_0013152170) was used as the outgroup. The robustness of the tree was re-appraised using the neighbour net method via SplitsTree6 ver 0.1.2-alpha [39]. The allelic clustering for each polymorphic block was conducted using a similar approach without the outgroup.

## 3. Results

### 3.1. Genetic Diversity and Natural Selection of pkmsp-1

**Figure 1:**
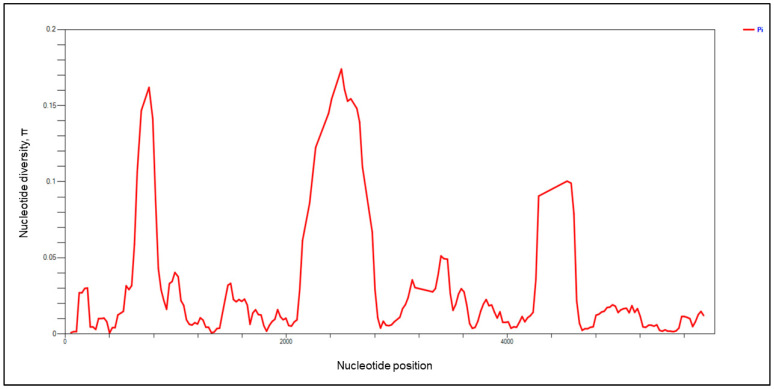
Sliding window plot (based on nucleotide diversity) of *pkmsp-1* with a window length of 100 bp and step size of 25 bp using DnaSP ver 6.12.

The organization of the full-length *pkmsp-1* gene obtained in this study was identical to previous reports [27,35], with five conserved blocks interspersed by four polymorphic blocks. There were 640 polymorphic sites, of which 127 were singleton and 456 were parsimony informative sites (Table 2). As a result of the extensive size variation within the polymorphic blocks, 805 sites with indels were identified. The nucleotide diversity in the sliding window plot (Figure 1) revealed high nucleotide diversity at the 5′ end, specifically at block II and block IV. Although *pkmsp-1* was more diverse (π = 0.026, Hd: 0.996) than other invasion-related proteins of *P. knowlesi*, the protein was under a strong negative/purifying selection (dN − dS = −5.87, *p* < 0.0001). Of the four polymorphic blocks, block IV contained the highest number of sites with indels (326 sites), followed by block VIII (244 sites), block VI (147 sites), and block II (74 sites), thus indicating that there were more gaps within blocks IV and VII than other polymorphic blocks. However, between the two polymorphic blocks with the highest indels, block IV (π = 0.174) was more polymorphic than block VIII (π = 0.100). Block II (π = 0.162) had a slightly lower nucleotide diversity than block IV, while, among the four polymorphic blocks, block VI demonstrated the lowest nucleotide diversity (π = 0.051). Overall, the polymorphic blocks were shown to be under positive/diversifying selection, whereas the conserved blocks were under negative/purifying selection. Tajima’s D, Fu and Li’s D*, and Fu and Li’s F* tests revealed that, overall, *pkmsp-1* did not depart significantly from neutrality.

### 3.2. Phylogenetic Analysis of pkmsp-1

**Figure 2:**
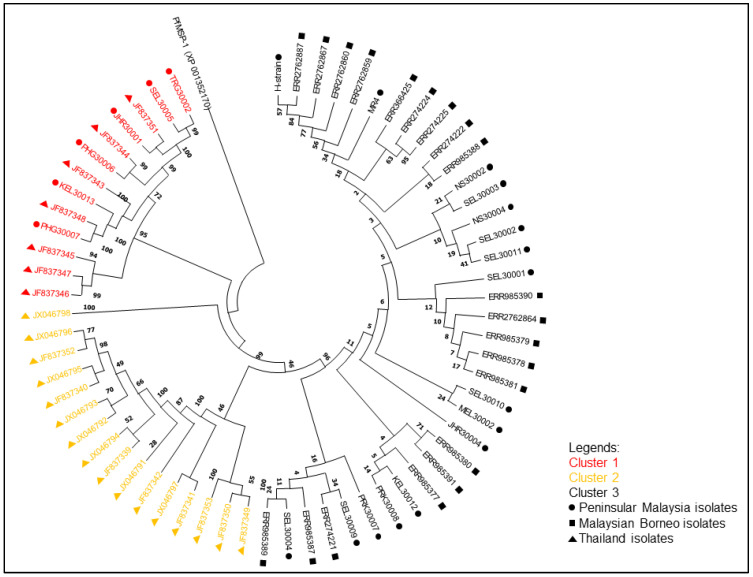
Neighbour-joining tree of 65 *pkmsp-1* sequences with 1000 bootstraps. The numbers next to the nodes represent the percentages that support the bifurcation for 1000 bootstrap replicates. The *pfmsp-1* was utilized as the outgroup.

**Figure 3:**
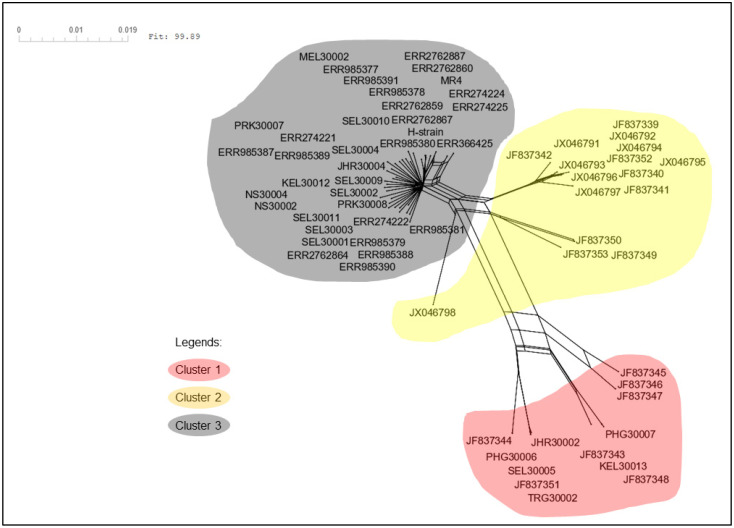
Neighbour-net tree of the same 65 sequences used in the neigbour-joining tree with a sum of differences goodness of fit = 99.89%. Three clusters were formed, and this corroborates with the clusters formed via the neighbour-joining method. Each cluster is denoted by a coloured bubble.

**Figure 4:**
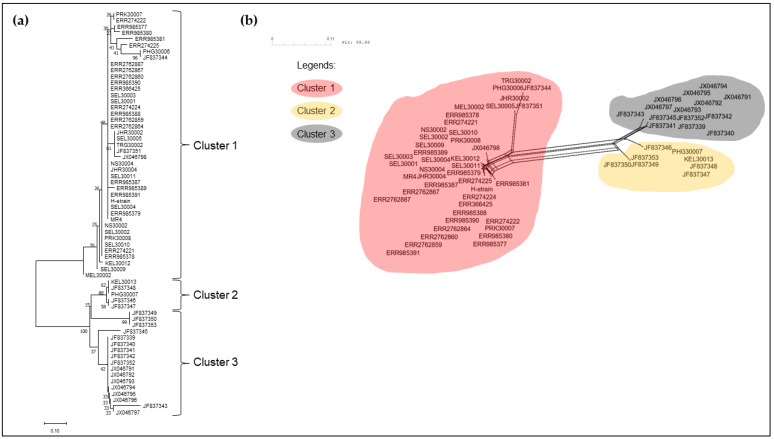
(**a**) Neighbour-joining tree of 65 sequences based on block II of *pkmsp-1* gene with 1000 bootstraps. The numbers next to the nodes represent the percentages that support the bifurcation for 1000 bootstrap replicates. (**b**) Neighbour-net tree of the same 65 sequences used in the neigbour-joining tree with a sum of differences goodness of fit = 99.96%. Three clusters were formed and corroborate with the clusters formed via the neighbour-joining method. Each cluster is denoted by a coloured bubble. Overall, three clusters are observed within block II.

**Figure 5:**
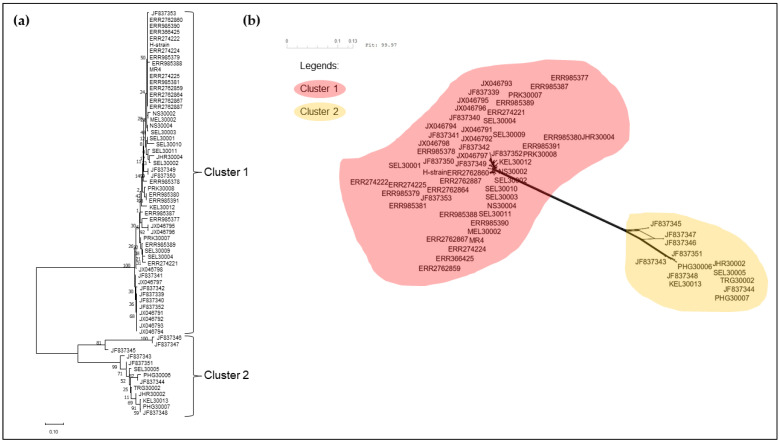
(**a**) Neighbour-joining tree of 65 sequences based on block IV of *pkmsp-1* gene with 1000 bootstraps. The numbers next to the nodes represent the percentages that support the bifurcation for 1000 bootstrap replicates. (**b**) Neighbour-net tree of the same 65 sequences used in the neighbour-joining tree with a sum of differences goodness of fit = 99.97%. Two clusters were formed and corroborate with the clusters formed via the neighbour-joining method. Each cluster is denoted by a coloured bubble. Overall, two clusters are observed within block IV.

**Figure 6:**
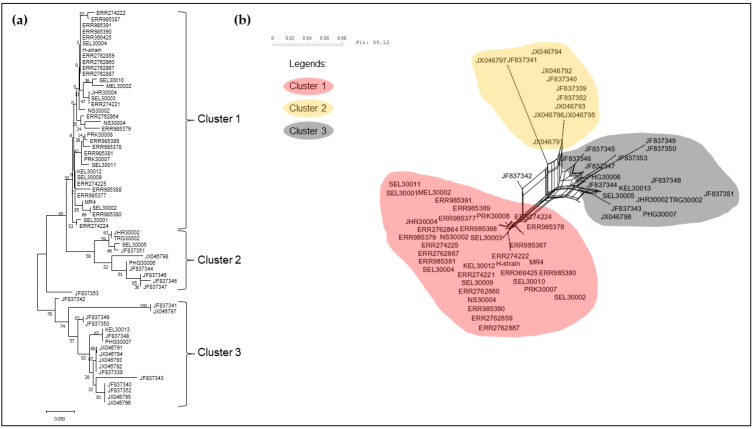
(**a**) Neighbour-joining tree of 65 sequences based on block VI of pkmsp-1 gene with 1000 bootstraps. The numbers next to the nodes represent the percentages that support the bifurcation for 1000 bootstrap replicates. (**b**) Neighbour-net tree of the same 65 sequences used in the neigbour-joining tree with a sum of differences goodness of fit = 99.12%. Three clusters were formed and corroborate with the clusters formed via the neighbour-joining method. Each cluster is denoted by a coloured bubble. Overall, three clusters are observed within block VI.

**Figure 7:**
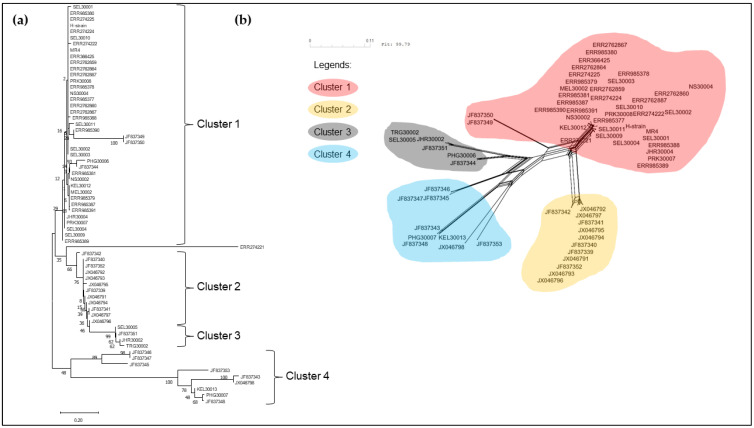
(**a**) Neighbour-joining tree of 65 sequences based on block VIII of *pkmsp-1* gene with 1000 bootstraps. The numbers next to the nodes represent the percentages that support the bifurcation for 1000 bootstrap replicates. (**b**) Neighbour-net tree of the same 65 sequences used in the neighbour-joining tree with a sum of differences goodness of fit = 99.96%. Four clusters were formed and corroborate with the clusters formed via the neighbour-joining method. Each cluster is denoted by a coloured bubble. Overall, four clusters are observed within block VIII.

The neighbour-joining phylogenetic tree of the pkmsp-1 sequences revealed three clusters (Figure 2). Cluster 3 comprised an admixture of Peninsular Malaysia and Malaysian Borneo sequences with no apparent sub-clustering. On the contrary, cluster 2 bifurcated from cluster 3, and contained Thailand sequences only. A few Peninsular Malaysia sequences were clustered together with some Thailand sequences in cluster 1. A similar clustering pattern was observed when the neighbour net method was employed (Figure 3). Further investigation of the polymorphic blocks (Figure 4, Figure 5, Figure 6 and Figure 7) revealed three allelic clusters for block II, two allelic clusters for block IV, three allelic clusters for block VI, and four allelic clusters for block VIII. In each block, the clusters obtained via the neighbour-joining and neighbour-net were similar. It is noteworthy that, in block IV, the two clusters bifurcated distinctly based on the neigbour-net method with shorter branches within the two clusters when compared with the neighbour-net tree of other polymorphic blocks. Meanwhile, the bifurcation of block VIII was most extensive with long branches linking the sequences, resulting in the highest number of clusters. As for block II, the bifurcation was as distinct as block IV, with cluster 3 forming a larger cluster upon bifurcation from cluster 2, thereby making cluster 3 the major allele in block II. The branch length between the clusters in block VI was shortest, thus indicating minute differences in sequences within the same cluster.

## 4. Discussion

This is the first study describing the genetic diversity of the polymorphic blocks of *pkmsp-1* with the inclusion of recent isolates from Peninsular Malaysia. The findings in this study are in contrast to a previous observation by Ahmed et al. (2018) [27], who reported that block VIII had the highest nucleotide diversity (π ≈ 0.290). The difference in nucleotide diversity distribution between these studies may be attributed to the number of sequences analysed, i.e., 65 in this study versus 11 in the study by Ahmed et al. (2018) [27]. The polymorphic blocks of *pkmsp-1* could serve as useful markers to genotype circulating *P. knowlesi* strain in a locality [35]. In addition to the two allelic families identified (based on the phylogenetic inference), block IV had the highest number of indels among the polymorphic blocks with the highest number of nucleotides. The indels are indicative of gaps within the block; hence, block IV may serve as a promising size polymorphism marker for the MOI of *P. knowlesi* malaria.

It is well established that there are three allelic families designated for *pfmsp-1* (RO33, MAD20, and K1) and two for *pfmsp-2* (FC27 and 3D7/IC) [31]. We propose to designate the T1 allelic family (corresponding to cluster 1 of block IV) and T2 allelic family (corresponding to cluster 2 of block IV) as genotyping the parasite allele circulating in a population, and, subsequently, calculating the multiplicity of infections (MOIs) are paramount. The MOI serves as a surrogate determinant of transmission intensity [40,41,42], where a higher mean MOI indicates high transmission intensity in the locality and vice versa. Genotyping is also useful in the context of ascertaining the parasite population. Previously, the K1 of *pfmsp-1* and the 3D7/IC of *pfmsp-2* were found to be predominantly circulating in Bobo-Dioulasso, Burkina Faso [41]. This suggests that *P. falciparum*, with either of those two alleles, adapts better in the population and is less virulent. Conversely, a study in Aceh, Indonesia revealed that the multiclonal infection by K1 + RO33 allelic families was strongly associated with a severe form of falciparum malaria [43]. In addition to testing the usefulness of the block IV of *pkmsp-1* as a genotyping marker for *P. knowlesi,* future studies may also investigate the association of the allelic families in block IV with disease severity. The T1 of block IV was the major allele or the predominant allelic family found in this study. Although speculative, it can be postulated that *P. knowlesi* harbouring a T1 allelic family adapts better in the population. Further investigations employing adequate sample size are deemed necessary to validate this.

The nucleotide diversity of *pkmsp-1* (π = 0.026) was higher when compared to the other genes encoding invasion-related proteins of *P. knowlesi*, such as *pktrap* (π = 0.009) [44], *pkama-1* (π = 0.004) [45], *pkβII* (Peninsular Malaysia: π = 0.015, Malaysian Borneo: π = 0.007) [46], *PkγII* (π = 0.019) [47], *pkdbpαII* (π = 0.013) [48], *pktramp* (π = 0.009) [49], *pkspatr* (π = 0.0146) [50], and *pkrap1 (*π = 0.0167) [51]. The extensive sequence polymorphism in the four polymorphic blocks of *pkmsp-1* could play a role in causing the vast difference in overall nucleotide diversity, if not the differences in gene size. This study found no significant departure of *pkmsp-1* from neutrality, in contrast to the findings of a previous study [27]. Conversely, possibly due to the higher number of isolates examined, this study revealed a purifying/negative selection on *pkmsp-1* (dN − dS = −5.87, *p* < 0.0001). This strong negative selection suggests structural or functional constraints of the protein. The disparity between the findings in this study with the previous study could stem from the different sample sizes.

Three clusters of *pkmsp-1* were observed based on the full length of *pkmsp-1*. Interestingly, some of the Peninsular Malaysian *pkmsp-1* sequences were clustered with those from Thailand in cluster 1, which may suggest a common ancestral origin of *P. knowlesi*. The Thailand sequences within cluster 1 (n = 7) were from six *P. knowlesi* human isolates and one *Macaca nemestrina* isolate and were all from provinces (Yala and Narathiwat) neighbouring northern Peninsular Malaysia. The transmission of *P. knowlesi* is thus not confined by a man-defined border. Recently, human knowlesi malaria cases were reported across the Laos–Vietnam border [52], and this could perhaps occur in a similar manner as in this study. The clustering of the Peninsular Malaysia isolates with the Thailand isolates corroborates the allopatric speciation of *P. knowlesi* in Malaysia, as proposed by Divis and colleagues, (2017) [53]. A previous study on the clustering of *P. knowlesi* based on its normocyte binding protein xa (*pknbpxa*) also showed the bifurcation of Peninsular-Malaysia-derived isolates from two other clusters [54]. This could be due to the submergence of the Sunda plate at the end of the last ice age, thus separating Malaysian Borneo from Peninsular Malaysia [48]. The admixture of Peninsular Malaysia and Malaysian Borneo isolates within cluster 3 mirrors the clustering of *P. knowlesi* reported in previous studies [49,50,54].

With the escalation of human knowlesi cases in Malaysia, vaccine development should be more progressive as an alternative approach, in addition to rigorous vector control and monitoring efforts implemented by the government. Since the last decade, ample studies investigating the natural selection acting on the invasion-related proteins of *P. knowlesi* have been conducted to gain insight and rudimentary data for better vaccine development and formulation [44,45,46,47,48,49,50,51]. The *pkama-1* was deciphered previously to be an under purifying/negative selection [45,55]. Hence, this serves as a determinant of a vaccine candidate for human knowlesi malaria. Recently, Ng et al. (2023) found that rabbit-raised antibodies against the region II of *pkama-1* displayed significant differences when compared to the negative control group, with close to 50% invasion inhibition [56]. The findings by Muh and colleagues (2018) are similar but with a lower antibody concentration used [57]. Similar investigations toward other invasion-related proteins of *P. knowlesi* should be conducted and compared, as antibodies are critical in developing immunity to malaria. Of note, RTS, S/AS01, the first malaria vaccine, specifically for falciparum malaria, has been approved to be used in pilot areas of Ghana, Kenya, and Malawi as part of the routine immunization for children [58]. The vaccine for *P. knowlesi* can be designed, perhaps in a similar way, by fusing the recombinant protein with an immunogen, such as the Hepatitis B surface antigen. Alternatively, a multistage or multiantigen vaccine is another promising approach [59].

MSP-1 is a promising candidate not only for *P. knowlesi* [60], but also for *P. vivax* [61] and *P. falciparum* [62]. However, vaccine studies are hampered owing to the extensive polymorphism across the gene. This can be observed from this study whereby high nucleotide diversity was observed for the full-length *pkmsp-1* as a consequence of the four polymorphic blocks, thus, highlighting the functionality of investigating the genetic diversity and polymorphism of the vaccine candidates. Instead, the conserved blocks, particularly the 42-kDa subunit of PkMSP-1 (PkMSP-1_42_), was studied as a potential vaccine candidate. Recent findings revealed novel binding peptides against the C-terminal of PkMSP-1_19_ [63,64]. This further aids the vaccine development or peptide-based anti-malarial drug formulation, and it is in line with the objectives of the One Health initiative to intervene and mitigate the endemic zoonotic infection caused by *P. knowlesi* via activities, including vaccine development. This would subsequently provide better health and well-being to vulnerable communities, as environmental components, which are taken into account to mitigate the spread of the infection.

## 5. Conclusions

Three clusters of *pkmsp-1* were observed across Thailand and Malaysia, with one cluster unique for Thailand. The *pkmsp-1* was under purifying/negative selection, albeit it was more diverse than several genes encoding invasion-related proteins of *P. knowlesi*. Block IV of *pkmsp-1* may serve as a promising genotyping marker for the MOI study of *P. knowlesi* malaria. Therefore, further investigation should be conducted on block IV to test its feasibility as an MOI genotyping marker in humans, as well as for the macaque population in Southeast Asia.

## Figures and Tables

**Table 1:** Primers used to sequence the *pkmsp-1* gene.

Primer	Sequence 5′ to 3′	Annealing Temperature (°C)
PkMSP-1_F1	CGTTGGCCACTTTTAAG	56
PkMSP-1_R1	GCTTCCAACAAGGGTGTTGTCT	
PkMSP-1_F2	CCGGATAATGGAAGGCAACCAA	58
PkMSP-1_R2	AGGTCGTTGATCATGGTGTC	
PkMSP-1_F3	CTACTGAAGCAGTACGCAC	58
PkMSP-1_R3	CGTCTTCATCATTGCCGAACAG	
PkMSP-1_F4	TGAAGCAGAACCAGCAAC	56
PkMSP-1_R4	AATGTGCAGCCAAAGCC	

**Table 2:** Estimates of genetic diversity indices and natural selection of *pkmsp-1*.

Block	SNPs	Indels	No. of Haplotypes	Diversity		dN − dS	Tajima’s D	Fu and Li’s D *	Fu and Li’s F *
				Nucleotide (π)	Haplotype (Hd)				
I	10	0	44	0.014	0.969	−1.46 **	−0.083	−0.173	−0.166
II	46	74	53	0.162	1.000	0.34	1.656	1.708 *	2.019 *
III	37	5	49	0.014	0.988	−4.17 **	−0.793	−1.535	−1.489
IV	231	326	55	0.174	1.000	0.98	0.545	1.105	1.057
V	12	0	36	0.012	0.971	−0.56	−1.190	−1.778	−1.861
VI	112	147	49	0.051	0.942	0.86	0.636	0.826	0.389
VII	25	9	49	0.018	0.987	−3.82 **	−0.250	−1.114	−0.930
VIII	138	244	51	0.100	1.000	3.19 **	0.495	1.462	1.314
IX	29	0	48	0.009	0.988	−4.81 **	−1.085	−1.468	−1.580
Full length	640	805	57	0.026	0.996	−5.87 **	−0.093	−0.193	−0.183

Note: * indicates *p* < 0.02 and ** indicates *p* < 0.0001.

## Data Availability

The nucleotide sequences are available on the NCBI GenBank (accession number: ON926538-ON926551, ON926557-ON926562). All figures are in already in the main article.

## Supplementary Materials

The following supporting information can be downloaded at: https://www.mdpi.com/article/10.3390/tropicalmed8050285/s1, Table S1: Details of Peninsular Malaysia specimens included in the study.
