# Supplementary material for: Inter-Population Genetic Diversity and Clustering of Merozoite Surface Protein-1 (pkmsp-1) of Plasmodium knowlesi Isolates from Malaysia and Thailand
Source: Trop Med Infect Dis. 2023 May 20;8(5):285. doi: 10.3390/tropicalmed8050285 (PMC10223053; doi:10.3390/tropicalmed8050285)
Supplement: Supplementary file 1 [file tropicalmed-08-00285-s001.zip › tropicalmed-2353504-supplementary.pdf]

Supplemental Table S1

Table S1. Details of peninsular Malaysia specimens included in the study.

No.	Specimen number	Origin	Accession number
1	KEL30012	Kelantan	ON926538
2	JHR30004	Johor	ON926539
3	NS30002	Negeri Sembilan	ON926540
4	NS30004	Negeri Sembilan	ON926541
5	SEL30001	Selangor	ON926542
6	SEL30002	Selangor	ON926543
7	SEL30003	Selangor	ON926544
8	PRK30007	Perak	ON926545
9	PRK30008	Perak	ON926546
10	SEL30004	Selangor	ON926547
11	SEL30009	Selangor	ON926548
12	SEL30010	Selangor	ON926549
13	SEL30011	Selangor	ON926550
14	MEL30002	Melaka	ON926551
15	JHR30001	Johor	ON926557
16	PHG30006	Pahang	ON926558
17	PHG30007	Pahang	ON926559
18	SEL30005	Selangor	ON926560
19	KEL30013	Kelantan	ON926561
20	TRG30002	Terengganu	ON926562
